# The Impact of Graphene and Diatomite Admixtures on the Performance and Properties of High-Performance Magnesium Oxychloride Cement Composites

**DOI:** 10.3390/ma13245708

**Published:** 2020-12-14

**Authors:** Anna-Marie Lauermannová, Filip Antončík, Michal Lojka, Ondřej Jankovský, Milena Pavlíková, Adam Pivák, Martina Záleská, Zbyšek Pavlík

**Affiliations:** 1 Department of Inorganic Chemistry, Faculty of Chemical Technology, University of Chemistry and Technology, Technická 5, 166 28 Prague, Czech Republic; Anna-Marie.Lauermannova@vscht.cz (A.-M.L.); filip.Antoncik@vscht.cz (F.A.); michal.Lojka@vscht.cz (M.L.); ondrej.jankovsky@vscht.cz (O.J.); 2 Department of Materials Engineering and Chemistry, Faculty of Civil Engineering, Czech Technical University in Prague, Thákurova 7, 166 29 Prague, Czech Republic; milena.pavlikova@fsv.cvut.cz (M.P.); adam.pivak@fsv.cvut.cz (A.P.); martina.zaleska@fsv.cvut.cz (M.Z.)

**Keywords:** composites, magnesium oxychloride, sorel cement, graphene, diatomite

## Abstract

A high-performance magnesium oxychloride cement (MOC) composite composed of silica sand, diatomite powder, and doped with graphene nanoplatelets was prepared and characterized. Diatomite was used as a 10 vol.% replacement for silica sand. The dosage of graphene was 0.5 wt.% of the sum of the MgO and MgCl_2_·6H_2_O masses. The broad product characterization included high-resolution transmission electron microscopy, X-ray diffraction, X-ray fluorescence, scanning electron microscopy and energy dispersive spectroscopy analyses. The macrostructural parameters, pore size distribution, mechanical resistance, stiffness, hygric and thermal parameters of the composites matured for 28-days were also the subject of investigation. The combination of diatomite and graphene nanoplatelets greatly reduced the porosity and average pore size in comparison with the reference material composed of MOC and silica sand. In the developed composites, well stable and mechanically resistant phase 5 was the only precipitated compound. Therefore, the developed composite shows high compactness, strength, and low water imbibition which ensure high application potential of this novel type of material in the construction industry.

## 1. Introduction

The overall amount of greenhouse gas (GHG) emissions released during the production of Portland cement (PC) accounts for 5–7% of all global emissions [1]. Predictions show, that by 2050, this amount will be 1.7–2.3 times bigger, if the production of PC is to continue at the same rate as it is going nowadays [2]. The released amount of CO_2_ and other GHG can be mainly attributed to the combustion of fuels whereby the necessary sintering temperature (~1450 °C) is reached. During the sintering itself, a large amount of CO_2_ is released because of the decomposition of limestone [3,4]. This growing amount of released GHG emissions trend is behind the increasing interest in finding an alternative to PC which is more ecologically sustainable. This approach offers the possibility of partially replace PC or its components by eco-friendly materials. As a partial replacement, waste materials such as ceramic or porcelain waste, fly ash, tire rubber and others are mostly studied [5,6,7,8]. Another possibility is to develop an ecologically sustainable material based on raw materials whose calcining temperature is lower than that of calcite. In this search reactive magnesia-based materials show great promise.

Magnesium oxychloride cement (MOC), discovered in 1867 [9], is an alternative reactive magnesia-based cement [10]. Generally, it can be described as a compound of the system MgO-MgCl_2_-H_2_O and there are four known phases of this sort of material which differ depending on the stoichiometric ratio between magnesium oxide, magnesium chloride and water. At ambient temperature, Phase 3 (3Mg(OH)_2_·MgCl_2_·8H_2_O) and Phase 5 (5Mg(OH)_2_·MgCl_2_·8H_2_O) are formed. At temperatures above 100 °C, Phase 2 (2Mg(OH)_2_·MgCl_2_·4H_2_O and 2Mg(OH)_2_·MgCl_2_·5H_2_O) and Phase 9 (9Mg(OH)_2_·MgCl_2_·4H_2_O) are present [11,12,13,14,15]. MOC has several unique properties and in some aspects such as resistance to abrasion, fire resistance, low thermal conductivity and mechanical properties, it can be superior to the commonly used Portland cement [16,17,18,19,20,21,22].

Graphene and other graphene derivatives belong to the group of carbon-based nanomaterials [23,24,25]. Graphene-based materials are two-dimensional sheets of carbon with a honeycomb structure [26]. These materials show unique electronic [27,28], optical [29,30,31], thermal [32,33,34] and mechanical properties [35,36,37] which make them applicable in many ways. The use of graphene in construction materials as an additive has been previously studied, with the results showing its positive impact on mechanical, thermal and electric properties and also the overall durability of the material [38,39,40].

Another distinct advantage in comparison to PC is the bonding ability to the wide range of fillers, due to its unique microstructure. It is known, that fillers such as silica glass [41], fuel ash [42], sawdust [42,43], asbestos waste [43] and many others, can be used in MOC-based composites while improving the properties of the final material, or at least not impacting them negatively in a meaningful way, in comparison to PC. This fact further improves the ecological aspects of MOC production. While PC can also use some waste materials as fillers (as mentioned in the first paragraph), comparatively, both the amount of the filler as well as filler themselves are significantly more restricted compared to MOC. However, the usage of larger filler content is not without its drawbacks. As an example, while the usage of fly ash in MOC as a way of disposing of unwanted waste material is desired from ecological point of view, it has to be carefully considered. It is well-known that the addition of fly ash to MOC reduces especially the compressive strength [44]. This effect is a function of fly ash content, so the material with a very high percentage of fly ash needs to be used in applications, where the lack of compressive strength is not a hindrance.

Diatomite can be described as a mineral formed in the process of sedimentation of the fragments of the carapace of diatom algae. It is a pale-colored, lightweight material mainly composed of the phase SiO_2_·nH_2_O, so it can be described as a silica-bearing material [44,45,46]. The material is abundant in various areas of the world and has been studied and characterized previously in the literature. It can be applied as a substrate for the synthesis of carbon-based nanomaterials [47] or as a material for the removal of heavy metals from water [48,49,50]. The low bulk density, high absorptive capacity, high surface area, and relatively low abrasion of diatomite make the material applicable as a partial filler replacement in construction materials. The use of diatomite as a partial replacement of Portland cement in cement admixtures has been previously described in the literature, showing the effect of diatomite on the mechanical properties, such as compressive strength, flexural strength, Young’s modulus, and water absorption [51,52,53,54].

In this paper, a composite material based on MOC and silica sand with graphene additive and diatomite powder was prepared and characterized using various analytical methods. Such material composition is unique and based on our analysis no similar material has been reported in the literature up to now. Diatomite was used as a partial sand substitute. A reference sample containing only MOC and silica sand was also prepared and used for comparison. The samples were analyzed in terms of their phase and chemical analysis using X-ray diffraction and energy dispersive spectroscopy. Optical microscopy was used to analyze the microstructures of all the samples. All of the samples were subjected to mechanical tests to show their compressive strength, flexural strength and elastic moduli. Ability to transport and accumulate water was characterized by the measurement of hygric parameters, porosity and pore size analysis. The thermal parameters of composites were the subject of the investigation as well.

## 2. Materials and Methods

**Figure 1:**
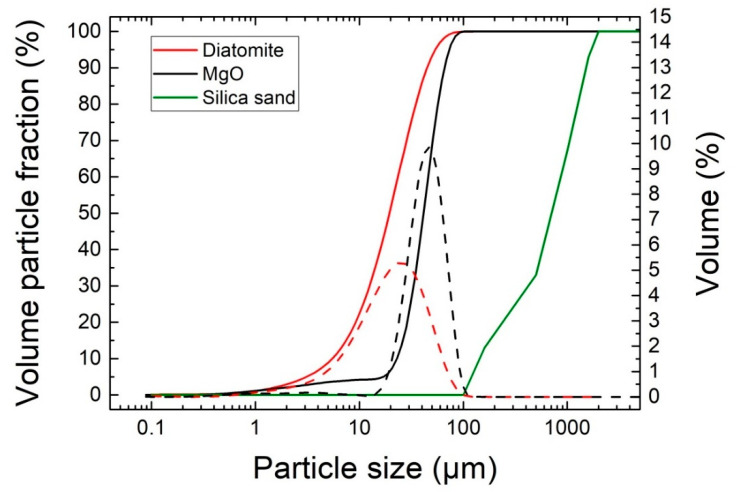
Particle size distribution of diatomite, MgO, and silica sand.

The light burnt magnesia (MgO) was a product of Styromagnesit Steirische Magnesitindustrie Ltd. (Oberdorf, Austria). A hydrous solution of MgCl_2_·6H_2_O (p.a. purity) delivered by Lachner s.r.o. (Neratovice, Czech Republic) had density of 26 Bé°. The 0–2 mm fraction of silica sand (Filtrační písky, spol. s r.o., Chlum u Doks, Czech Republic) was used as only filler in reference samples. The loose bulk density of sand used was 1678 kg·m^−3^. In modified composite mix, fine-grained diatomite (Blaine fineness 2087 m^2^·kg^−1^) produced in LB MINERALS s.r.o., (Horní Bříza, Czech Republic) was used as 10% volumetric replacement of silica sand. The grain size analysis of quartz sand was done by sieve method in accordance with the EN 933-1 [55]. The particle size distribution of MgO and diatomite was investigated on a laser diffraction principle using an Analyssete 22 MicroTec plus apparatus (Fritsch, Idar-Oberstein, Germany). The particle size of the used materials is apparent from Figure 1. Both diatomite and MgO exhibited unimodal particle size distribution with maxima at 26.8 and 46.2 μm respectively.

**Figure 2:**
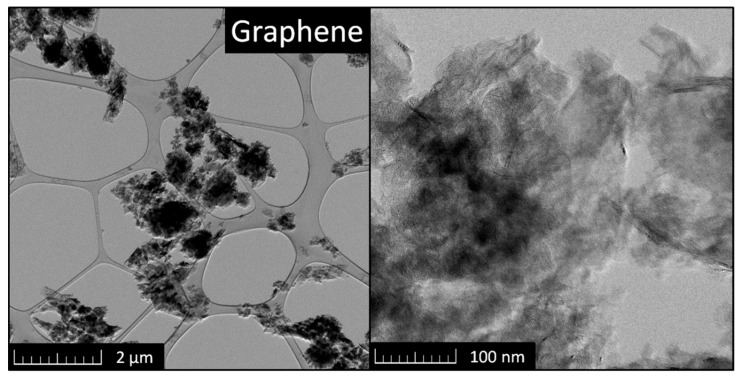
The HR-TEM scan of graphene.

The graphene nanoplatelets having declared surface area 500 m^2^·g^−1^ were provided by Alfa Aesar (Thermo Fisher Scientific, Kandel, Germany). The purity of graphene used was 99.9 wt.% and negligible traces of other elements such as S, Si, and Fe were identified. The microstructure of the graphene was studied by HR-TEM (Jeol, Tokyo, Japan). The TEM data presented in Figure 2 prove a characteristic layered structure of graphene and a thickness of a few atoms.

A magnesium chloride solution of required concentration was prepared using MgCl_2_·6H_2_O and tap water. Part of the solution was used for the dispersion of graphene nanoplatelets. For this purpose, the magnesium chloride solution with graphene was first sonicated in ultrasonic bath for 15 min and then dispersed for 5 min using a T18 UltraTurrax (IKA, Staufen im Breisgau, Germany) operating at 7000 rpm. The resulting suspension was added to MgO powder and mixed for 90 s. After that the sand or sand/diatomite mix were added and the mixture was stirred for another 90 s.

The fresh composite was then poured in two layers into prismatic iron molds having dimensions of 160 mm × 40 mm × 40 mm and compacted on a vibrating table. The specimens were demolded after 24 h, and then they were cured for 27 days in laboratory at temperature of (23 ± 2) °C and relative humidity of (50 ± 5)%. D The composition of investigated composites is presented in Table 1. The dosage of graphene nanoplatelets was 0.5 wt.% of the sum of MgO and MgCl_2_·6H_2_O mass.

The graphene nanoplatelets were subjected to the HR-TEM. For that purpose, an EFTEM 2200 FS microscope (Jeol, Tokyo, Japan) was applied.

XRD data were collected at room temperature on a D8 Phaser powder diffractometer (Bruker, Karlsruhe, Germany) with parafocusing Bragg–Brentano geometry using CuKα radiation (λ = 0.15418 nm, *U* = 30 kV, *I* = 10 mA).

The morphology was investigated using SEM with a FEG electron source (Tescan Lyra dual beam microscope, Tescan Brno, s.r.o., Brno, Czech Republic). EDS was measured by an X-Max^N^ analyser equipped with a 20 mm^2^ SDD detector (Oxford Instruments, Abingdon, UK) and AZtecEnergy software (v. 3.0).

Optical microscopy of composite samples was performed by a Navitar macro-optics microscope (Rochester, NY, USA) with optical zoom up to 110× and recorded with 2/3” digital camera (Sony, Minato, Japan) having a resolution of 5 Mpix. The sample was illuminated by a white LED ring light source with individually addressable segments and intensity. NIS-Elements BR 5.21.02 software with an Extended Depth of Focus Module (EDF) was used for imagining and analysis of the samples.

For the 28-days matured composites, structural, micro-structural, mechanical, hygric, and thermal parameters were determined. Except for the MIP test, five samples of each material were tested. The presented data represents mean value calculated based on the results obtained for the particular samples. Where applicable, the expanded combined uncertainty of the presented data was given.

Basic characterization of the hardened composites was done using the bulk density, specific density and total open porosity assessment. The bulk density test was conducted in compliance with the standard EN 1015-10 [56]. For the specific density measurement, an Pycnomatic ATC apparatus (Porotec, Hofheim, Germany) operating on a helium pycnometry principle was used. The total open porosity was calculated based on the specific density and bulk density values, as originally presented, e.g., in [57]. Pore size distribution was investigated by mercury intrusion porosimeters of Pascal series (Thermo Fisher Scientific, Waltham, MA, USA). The typical sample mass was ~2 g.

Mechanical resistance and stiffness were characterized by flexural strength *f*_f_ (MPa), compressive strength *f*_c_ (MPa), and dynamic modulus of elasticity *E*_d_ (GPa). The strength tests were realized as prescribed in the EN 1015-11 [58]. A Vikasonic apparatus (Schleibinger Geräte, Buchbach, Germany) was used for recording of ultrasound wave velocity and evaluation of the dynamic modulus of elasticity.

The poor resistance of MOC-based materials against moisture damage is reported in the literature [59,60]. Therefore, the parameters that define the water transport and storage were the subject of the experimental analysis. The maximum capillary water absorption *W*_a_ (%) and 24-h water absorption *W*_a24_ (%) were measured according to the EN 13755 [61]. The water absorption coefficient *A*_w_ (kg·m^−2^·s^−1/2^) and apparent moisture diffusivity *κ*_app_ (m^2^·s^−1^) were evaluated based on the free water intake experiment. This test was conducted as introduced in the EN 1015-18 [62], and the data were assessed as proposed by Feng et al. [63].

Among thermophysical parameters, thermal conductivity, thermal diffusivity, and volumetric heat capacity were tested. They were examined using a Hot Disk TPS 1500 thermal constants analyzer (Hot Disk AB, Göteborg, Sweden) operating on a transient plane source technique [64]. The tests were conducted on dry samples at laboratory temperature of (23 ± 2) °C.

## 3. Results and Discussion

**Figure 3:**
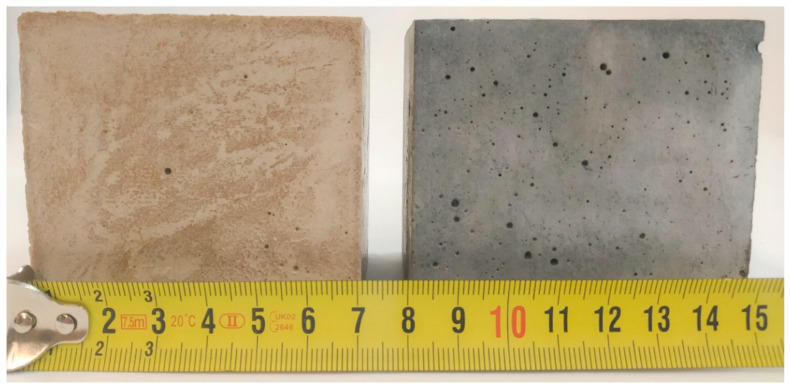
The prepared composites: MOC-REF (left), MOC-DG (right).

In this study, the impact of graphene and diatomite addition to the magnesium oxychloride matrix was investigated. Prepared high-performance composites are shown in Figure 3. Samples were termed MOC-REF (REFerence sample of Magnesium Oxychloride Cement) and MOC-DG (Magnesium Oxychloride Cement with Diatomite and Graphene).

**Figure 4:**
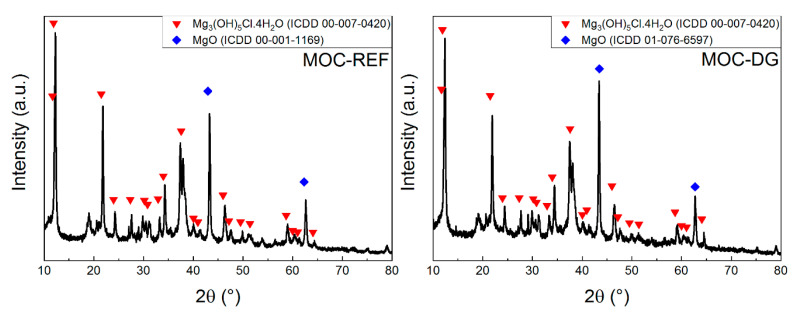
The diffraction patterns of samples MOC-REF and MOC-DG.

The phase composition of both samples was studied using X-ray Diffraction. The XRD analysis was conducted on paste samples in order to avoid very strong reflections of quartz. Usually, when sand is present in the samples, the quartz is the only visible phase. Both diffraction patterns can be seen in Figure 4. The results show the presence of the phase Mg_3_(OH)_5_Cl·4H_2_O (ICDD 00-007-0420) and MgO (ICDD 00-001-1169). The diatomite as well as graphene are not visible in MOC-DG sample due to amorphous nature of diatomite and very low graphene content.

**Figure 5:**
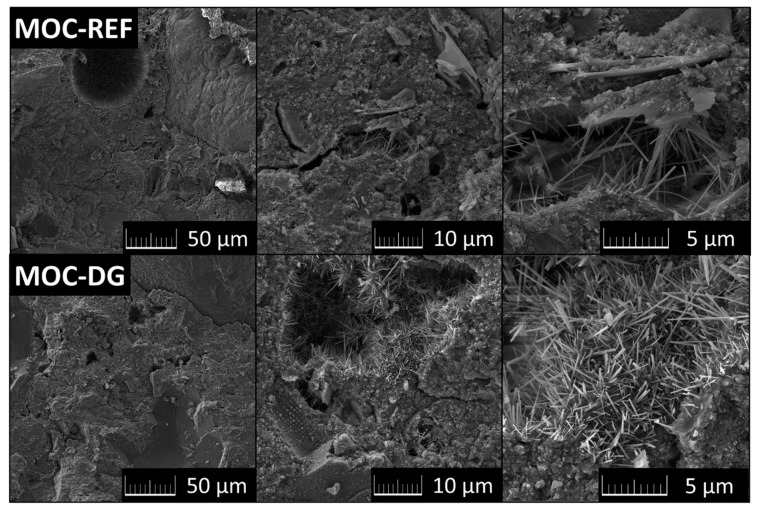
SEM micrographs of MOC-REF and MOC-DG.

The morphology of the composites was studied by SEM (see Figure 5). Highly compact structure was detected. If any defects were observed (bubbles, cracks), then these areas were inter-grown by needles from the MOC phase 5, as can be clearly visible from the SEM micrographs. This needle-like shape is typical for MOC phase 5. The typical dimensions of such needles are 1–10 μm in length and a few hundreds of nanometers in width. Also, the diatomite and graphene are well connected with MOC binder.

**Figure 6:**
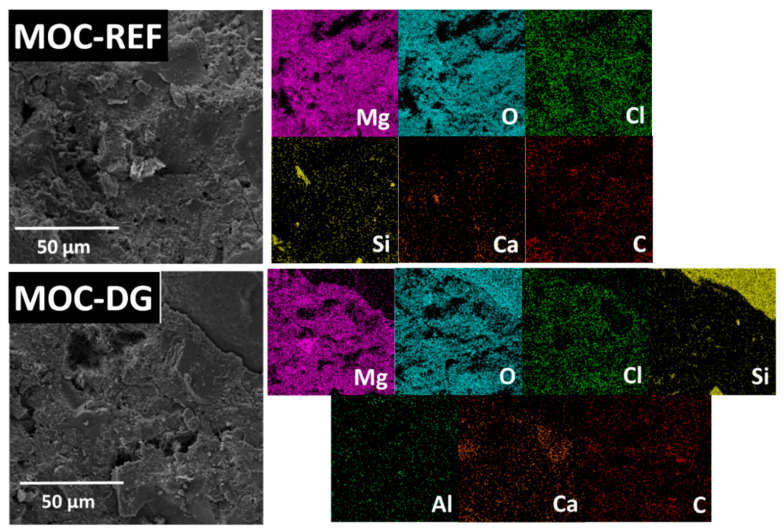
The elemental maps of samples MOC-REF and MOC-DG.

To determine the chemical composition of the composites, EDS was used. The qualitative analysis showed the presence of the following major elements: magnesium, oxygen, carbon, chlorine, calcium, silicon and aluminum, whose elemental maps can be seen in Figure 6.

The quantitative analysis results are shown in Table 2. The presence of carbon is caused by MOCs ability to absorb CO_2_. The presence of calcium and iron is caused by the low amount of impurities present in the raw materials, namely the caustic MgO.

**Figure 7:**
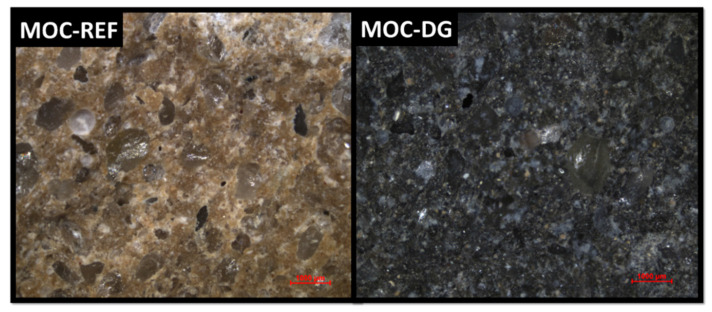
Images obtained by Optical Microscopy of samples MOC-REF and MOC-DG. Scale bar is 1000 μm.

The microstructure of both samples was analyzed using optical microscopy (see Figure 7). Both MOC-REF and MOC-DG showed compact structure without any visible defects. Silica sand is well distributed in the MOC matrix. Even a very low graphene content significantly change the color of the composite.

The basic structural properties of the analyzed composites are presented together with the mechanical parameters in Table 3. Taking into consideration the principles of the applied porosity assessment methods, the porosity values obtained from the combined gravimetric/pycnometric measurement and data provided by the mercury intrusion porosimetry (*P*_Hg_), were almost similar. The use of diatomite and graphene nanoplatelets led to the high drop in porosity which was due to the low dimension of diatomite particles and graphene agglomerates.

**Figure 8:**
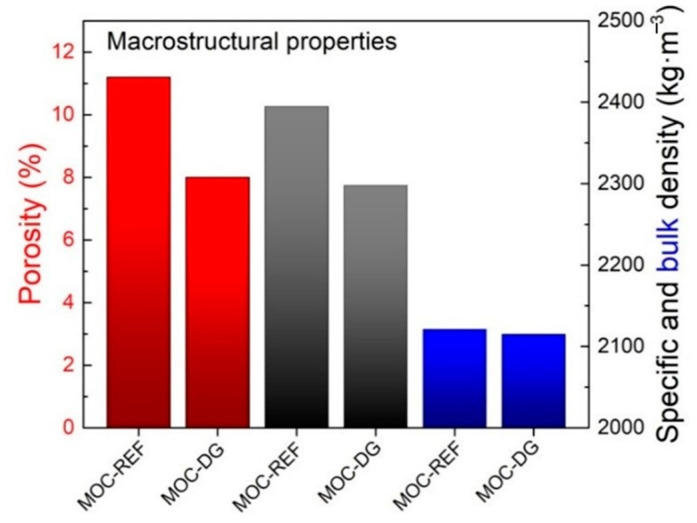
Reduction of the macrostructural parameters of MOC-REF and MOC-DG composites.

Because of the lower specific density of diatomite than that of silica sand, both the bulk density and specific density of MOC-DG material were slightly reduced. The drop of the investigated macrostructural parameters is well apparent from Figure 8. 

Consistent with the decrease in porosity, the mechanical strength of MOC-DG composite was greatly improved. It was the result of the three mutually acted effects: (i) low porosity, (ii) activation of two-dimensional graphene nanoplatelets that bridged the gaps between the Phase 5 needles, unreacted MgO, and diatomite particles, (iii) high hardness, compressive strength, and flexural of graphene nanoplatelets [65,66,67]. The biggest improvement in the tested mechanical parameters by the synergic action of diatomite and graphene was achieved for the compressive strength, which increased for MOC-DG of approx. 30% compared to the reference composite MOC-REF. However, also the flexural strength and dynamic elastic modulus were moderately enhanced.

**Figure 9:**
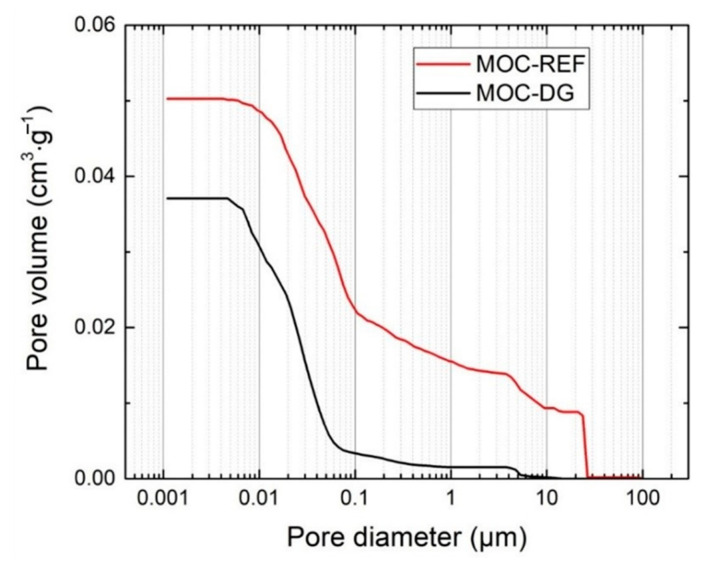
Cumulative pore volume of MOC-REF and MOC-DG composites.

**Figure 10:**
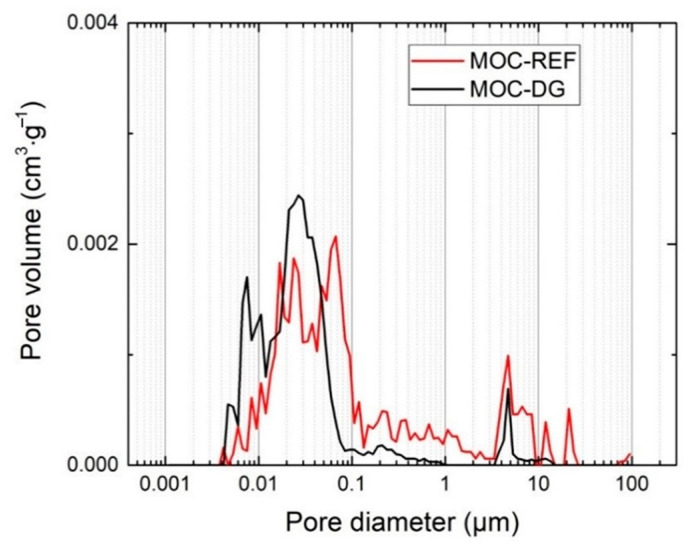
Incremental pore volume distribution of MOC-REF and MOC-DG composites.

The pore size distribution measured by mercury porosimetry is shown in Figure 9 and Figure 10. Both the cumulative and incremental pore volume distribution curves gave evidence of the improved packing and consolidation of MOC-DG material compared to the reference one. The total pore volume dropped from 0.05038 cm^3^·g^−1^ (MOC-DG) to 0.03721 cm^3^·g^−1^ (MOC-REF). Accordingly, the average pore size decreased from 0.0412 to 0.0174 μm.

The effect of the use of graphene and diatomite on the hygrothermal performance of the newly developed composite is evident from Table 4. The water absorption, water absorption coefficient, and moisture diffusivity were considerably lowered for the MOC-DG material compared to the reference one. The assessed hygric parameters thus well corresponded with the macrostructural properties and pore size distribution data. Quantitatively, the water absorption coefficient was low as typical capillary active materials have *A*_w_ value of about two orders of magnitude higher [68,69]. For example, the standard EN 998 [70] introduces three classes of rendering mortars waterproofing. In the W1 type and W2 type mortars the *A*_w_ values should be 0.4 and 0.2 kg·m^−2^·s^−1/2^ respectively [71]. It means the tested composites can be considered as waterproof in this manner. Typically, high dry thermal conductivity and thermal diffusivity were determined for both composites. The porous diatomite particles slightly reduced the thermal conductivity value of the MOC-DG composite and thus partially mitigated heat transport in its structure. On the other hand, the ability to store heat was almost unaffected by the incorporation of diatomite and graphene into composite mixture. The thermal properties or MOC-based composites were only scantly studied up to now. Certain exception represents work published by Xu at al. [72] who analyzed thermal performance of MOC composites with cenospheres. Authors received for the reference MOC composite with silica sand used a filler thermal conductivity of approx. 2.3 (W·m^−1^·K^−1^). Of course, it is lower value than obtained in our case, but their material had much higher porosity.

## 4. Conclusions

The impact of graphene and diatomite admixing on the performance and properties of MOC composites was studied. A broad test campaign which involved sophisticated and effective characterization analyses such as HR-TEM, XRD, XRF, SEM, and EDS were applied to investigate the raw materials and composites matured for 28-days. The data acquired from the conducted tests allows us to point out the following most substantial results:

- the identified crystalline phases in both composites were well stable and durable phase 5 (Mg_3_(OH)_5_Cl·4H_2_O) and unreacted MgO residue;
- the highly compacted structure of graphene-doped composite was identified, where possible defects were inter-grown by phase 5 needle like crystals;
- diatomite and graphene were well distributed and fixed in MOC matrix;
- the porosity, bulk density, and specific density were reduced by the use of graphene nanoplatelets and diatomite;
- the compressive strength of MOC-DG composite was greatly improved due to the high hardness and mechanical strength of graphene, lowered porosity, and activation of two-dimensional graphene-based reinforcement;
- as the average pore size was significantly reduced by the mutual action of diatomite and graphene in composite mixture, the water transport and accumulation was highly limited in MOC-DG materials which is very promising finding for its improved durability in the sense of moisture damage;
- the use of diatomite slightly reduced the thermal conductivity of the newly developed composite, but its heat transport properties remained high.

Summarizing the main findings of the study above listed, the diatomite/graphene enriched MOC composite represents an interesting alternative material that meets the current technical criteria and functional standards imposed on novel high-performance materials for construction industry. The acquired data will be used in the near future for the design of new construction composites designed for specific construction purposes and applications.

## Figures and Tables

**Table 1:** The dosage of the particular components in composite mixtures (g).

Composite	MgO	MgCl_2_·6H_2_O	Water	Silica Sand	Diatomite	Graphene
MOC-REF	584.4	258.9	215	3 × 497.7	-	-
MOC-DG	584.4	258.9	215	3 × 382.8	26.6	4.2

**Table 2:** Chemical Composition of MOC-REF and MOC-DG obtained by EDS.

Element	MOC-REF	MOC-DG
Mg	32.7	21.8
O	46.1	45.7
C	11.3	12.0
Cl	8.0	6.0
Ca	1.2	5.0
Si	0.7	9.2
Al	0.0	0.3

**Table 3:** Macrostructural and mechanical properties of the tested composites.

Material	*ρ*_s_ (kg·m^−3^)	*ρ*_b_ (kg·m^−3^)	*P* (%)	*P*_Hg_ (%)	*f*_f_ (MPa)	*f*_c_ (MPa)	*E*_d_ (GPa)
MOC-REF	2395 ± 29	2121 ± 30	11.2 ± 0.2	10.8	23.1± 0.3	67.3 ± 0.9	33.8 ± 0.8
MOC-DG	2298 ± 28	2115 ± 30	8.0 ± 0.2	8.11	25.6 ± 0.3	87.7 ± 1.2	37.5 ± 0.9

**Table 4:** Hygric and thermal parameters of the tested composites.

Parameter	MOC-REF	MOC-DG
*W*_a_ (%)	4.28	1.94
*W*_a24_ (%)	2.85	0.94
*A*_w_ (kg·m^−2^·s^−1/2^)	0.0061	0.0023
*κ*_ap*p*_ × 10^−11^ (m^2^·s^−1^)	4.90	3.47
*λ*_d_ (W·m^−1^·K^−1^)	3.270	3.151
*a*_d_ × 10^−6^ (m^2^·s^−1^)	2.112	2.154
*C*_vd_ × 10^6^ (J·m^−3^·K^−1^)	1.548	1.463
